# Japan’s State of Emergency: How Political Decisions Affected Post-COVID-19 Syndrome

**DOI:** 10.31662/jmaj.2023-0147

**Published:** 2024-06-10

**Authors:** Yudai Kaneda

**Affiliations:** 1 School of Medicine, Hokkaido University, Hokkaido, Japan

**Keywords:** post-coronavirus disease 2019 syndrome, sequelae, early pandemic stage, state of emergency, Japan

Kinugasa et al. reported the incidences of post coronavirus disease 2019 (COVID-19) syndrome in Japan, including nursing care requirements and its potential relationship with vaccination status and SARS-CoV2 strains ^(1)^. Although their study reveals valuable insights, understanding the classification based on political characteristics―rather than mere geographical positioning―has impacted the incidence of post-COVID-19 syndrome, especially for the early pandemic stage (Period 1) ^(1)^, and provided us further insights.

Indeed, a review of risk factors associated with the post acute sequelae of COVID-19 underscores the significance of social determinants of health, encompassing societal, political, and environmental facets and their subsequent implications for patients ^(2)^. Furthermore, an EHR-based cohort study has identified three primary domains of environmental risk factors for the post acute sequelae of COVID-19: the natural, built, and social environments ^(3)^.

Contextually, the classification method based on the emergency declaration timing proposed in our previous research can serve as a useful reference ^(4)^. In Japan, an emergency declaration was issued for seven prefectures on April 7, 2020, including Tokyo, Kanagawa, Saitama, Chiba, Osaka, Hyogo, and Fukuoka, collectively referred to as “Specific Alert Area A.” This declaration was expanded nationwide on April 16, 2020. Subsequently, six other prefectures―Hokkaido, Ibaraki, Ishikawa, Gifu, Aichi, and Kyoto―were added and designated as “Specific Alert Area B,” enhancing measures against the virus spread. However, by May 14, 2020, the declaration was lifted in 39 prefectures but remained effective for eight: Hokkaido, Tokyo, Saitama, Chiba, Kanagawa, Osaka, Kyoto, and Hyogo. Consequently, by May 25, 2020, the emergency status in these prefectures was revoked. Therefore, Japan’s state of emergency statement was declared based on the prevalence of infections and strains on medical systems rather than geographical characteristics. Specifically, during the initial analysis of the pandemic, the classification into Specific Alert Areas A and B and the rest offers crucial insights. Considerably, statistically significant differences have been reported in various facets, such as the cancellation or postponement of surgical operations and the movement of people, during analyses utilizing such classifications ^(4), (5)^.

Given these perspectives and findings, Kinugasa et al. are kindly invited to reflect upon and engage with further analysis based on the abovementioned points. The insights on the influence of political characteristics on post-COVID-19 syndrome in Japan would enrich the collective understanding of the broad implications of the disease.

## Article Information

### Conflicts of Interest

None

### Author Contributions

Conception and writing - original draft; YK
